# Modified host defence peptide GF19 slows TNT-mediated spread of corneal herpes simplex virus serotype I infection

**DOI:** 10.1038/s41598-024-53662-4

**Published:** 2024-02-19

**Authors:** Neethi C. Thathapudi, Natalia Callai-Silva, Kamal Malhotra, Sankar Basu, Mozhgan Aghajanzadeh-Kiyaseh, Mostafa Zamani-Roudbaraki, Marc Groleau, Félix Lombard-Vadnais, Sylvie Lesage, May Griffith

**Affiliations:** 1 Maisonneuve-Rosemont Hospital Research Centre, Montreal, QC H1T 2M4 Canada; 2 Department of Ophthalmology, Université de Montréal, Montreal, QC H3C 3J7 Canada; 3 Institute of Biomedical Engineering, Université de Montréal, Montreal, QC H3T 1J4 Canada; 4 Department of Microbiology, Asutosh College, (Affiliated With University of Calcutta), Kolkata, 700026 India; 5 Département de Microbiologie, Infectiologie et Immunologie, Université de Montréal, Montréal, QC H3T 1J4 Canada; 6 Present Address: Division of Cardiology, Department of Medicine, University of Ottawa Heart Institute, University of Ottawa, Ottawa, K1Y 4W7 Canada; 7 Present Address: Department of Cellular and Molecular Medicine, University of Ottawa, Ottawa, K1H 8M5 Canada

**Keywords:** Biotechnology, Medical research

## Abstract

Corneal HSV-1 infections are a leading cause of infectious blindness globally by triggering tissue damage due to the intense inflammation. HSV-1 infections are treated mainly with antiviral drugs that clear the infections but are inefficient as prophylactics. The body produces innate cationic host defence peptides (cHDP), such as the cathelicidin LL37. Various epithelia, including the corneal epithelium, express LL37. cHDPs can cause disintegration of pathogen membranes, stimulate chemokine production, and attract immune cells. Here, we selected GF17, a peptide containing the LL37 fragment with bioactivity but with minimal cytotoxicity, and added two cell-penetrating amino acids to enhance its activity. The resulting GF19 was relatively cell-friendly, inducing only partial activation of antigen presenting immune cells in vitro. We showed that HSV-1 spreads by tunneling nanotubes in cultured human corneal epithelial cells. GF19 given before infection was able to block infection, most likely by blocking viral entry. When cells were sequentially  exposed to viruses and GF19,  the infection was attenuated but not arrested, supporting the contention that the GF19 mode of action was to block viral entry. Encapsulation into silica nanoparticles allowed a more sustained release of GF19, enhancing its activity. GF19 is most likely suitable as a prevention rather than a virucidal treatment.

## Introduction

The cornea is the clear window at the front of the eye which plays a crucial role in vision by directing light onto the retina. Severe infections or injuries causing permanent opacity in the cornea may result in blindness. Herpes Simplex Virus serotype 1 (HSV-1) is a DNA virus, approximately 125 nm in diameter, that has evolved to replicate mainly in epithelial cells and neurons,^1^ such as those of the cornea. HSV-1 corneal infection is the leading cause of infectious blindness worldwide in both developed and developing regions, with 1 to 1.5 million new cases and 9 million recurrent ones annually^2^. Once a cornea is infected, the virus can be transported retrogradely through the cornea nerves to the trigeminal ganglia where it establishes latency^3^. Initial or primary infections commonly affect only the epithelium with minimal or no requirement for medical intervention. Viruses are cleared from the eye by effective antiviral drugs. However, these drugs do not prevent viral reactivation, which in turn causes an inflammatory cascade (keratitis) that can result in tissue damage.

Structurally, HSV-1 comprises a nucleocapsid containing viral DNA, a surrounding proteinaceous tegument layer, and an outermost lipid envelope with surface glycoproteins^4,5^. The capsid is involved in the retrograde transport of the virus in neurons and release of the viral genome into the host cell nucleus. It also facilitates the release of nascent virus from the host cell nucleus^6^. The tegument comprises over 20 proteins with essential roles in viral transport and maturation^7^. The outermost lipid coat is derived from the internal membrane of the infected host cells, where the membrane cholesterol is needed for critical fusion with host cells to enable viral entry^8^. Commonly prescribed antiviral drugs such as acyclovir or ganciclovir comprise synthetic nucleoside analogues that clear viral infections by blocking viral DNA polymerase and thereby preventing viral replication^9^; they do not act directly on the viruses. Severe HSV-1 infections due to high amounts of virus in the cornea can cause uncontrolled inflammation or keratitis resulting in cell death and scarring^1,2^.This in turn can lead to the cornea becoming thinner and opaque, increasing the risk for corneal perforation^1^. Prophylactic use of high doses of antiviral drugs does not block all recurrent disease^10^ and drug resistance has been reported^11^. Therefore, an antiviral compound that can prevent reactivation would be extremely beneficial.

Tunnelling nanotubes (TNTs) are thin cytoplasmic connections between cells of similar or different types, through which exchange of organelles, cytosolic proteins, nucleic acids, lipids etc., occur, forming an important part of direct long and short distance cell–cell communication^12^. They are transient structures and therefore, visualising them in vivo is extremely difficult. Recent studies reported their role in viral infection spread amongst cells. For example, the SARSCoV2 virus was reported to “hijack” TNT machinery to infect cells that lack the ACE receptors^13^. Bovine herpesvirus 1 (BoHV-1) also employs TNTs for viral spread, with viral particles seen passing through the tubes even in the presence of neutralising antibodies that blocked free viral entry into the cells^14^. TNTs in viral infections are relatively new observations and not much is known regarding their molecular mechanism. However, evidence shows that they have critical functions in long distance viral spread^12,15^. Here, we show that TNTs are involved in the HSV-1 infection of human corneal epithelial cells in vitro.

Cationic human defence peptides (cHDP) are small 10 to 150 amino acids long peptides, with a net charge between − 3 and + 20. Over 100 HDPs were identified in various human tissues and epithelial surfaces, including skin, eyes, ears, mouth, and gut, providing defence against pathogens including viruses, and also modulating inflammation^16^. Cathelicidins and defensins are two main cHDP families found at the sites of injury or infection where they cause disintegration of pathogen membranes, stimulate chemokine production, and attract immune cells. Humans have only one cathelicidin, LL37, which is produced by epithelial cells including corneal and conjunctival cells, and neutrophils. LL37 is derived from a larger precursor, hCAP-18 and its expression is upregulated during viral infection. LL37 possesses broad-spectrum antiviral activity, including activity against HSV-1, human papillomavirus, adenovirus, etc. It acts by disrupting the lipid membrane of the virus and preventing their replication, as observed in its action against influenza viruses^17^. Additionally, LL37 also induces the production of type I interferons, which are signalling molecules with key roles in activating the innate immune response against viruses^18–20^.

We previously tested LL37 in vitro against HSV-1*,* where it blocked viral entry into cultured HCECs, albeit not completely^21^. Despite the promising results, therapeutic use of LL37 is limited by its cytotoxicity and rapid degradation^22^. To overcome these, researchers have attempted to engineer LL37 with increased stability, reduced toxicity, and targeted specificity. We tested its smallest bioactive fragment, KR12, against HSV-1 in an infected rabbit corneal model. While KR12 was biocompatible, promoted epithelial healing and showed good antiviral activity in culture, its in vivo antiviral activity was weak^23^. KR12 released from silica nanoparticles (SiNP) delivered through an implantable biosynthetic cornea could not effectively block virus activity in the rabbits, but was able to nevertheless promote in situ corneal tissue regeneration^23^.

Our aim was to identify an innate cHDP with anti-HSV-1 activity that would not lead to side effects, being produced naturally in the body. We examined active LL37 fragments and predicted a plausible interaction model under hydrophilic biological conditions. A 17 amino-acid GF17 fragment of LL37 containing the KR12 sequence, but with extra amino acids G and F at the N-terminal and NLV at the C-terminal^24^, had effectively blocked Zika^25^ and HIV^26^ viruses. We added two cell penetrating amino acids, lysine and leucine (KL), to the C-terminal of GF17 to enhance its ability to penetrate viral membranes and compared the theoretical activity of this new GF19 fragment (GF-KRIVQRIKDFLR-NLV-KL) to LL37, and the original GF17. The peptides were then tested experimentally against HSV-1 viruses in vitro. We used the McKrae strain of HSV-1 isolated from an infected patient’s eye in our studies as it is well-characterized and clinically relevant for studying severe cornea viral infections^27^.

## Results

### HSV-1 infection of human corneal epithelial cells

**Figure 1:**
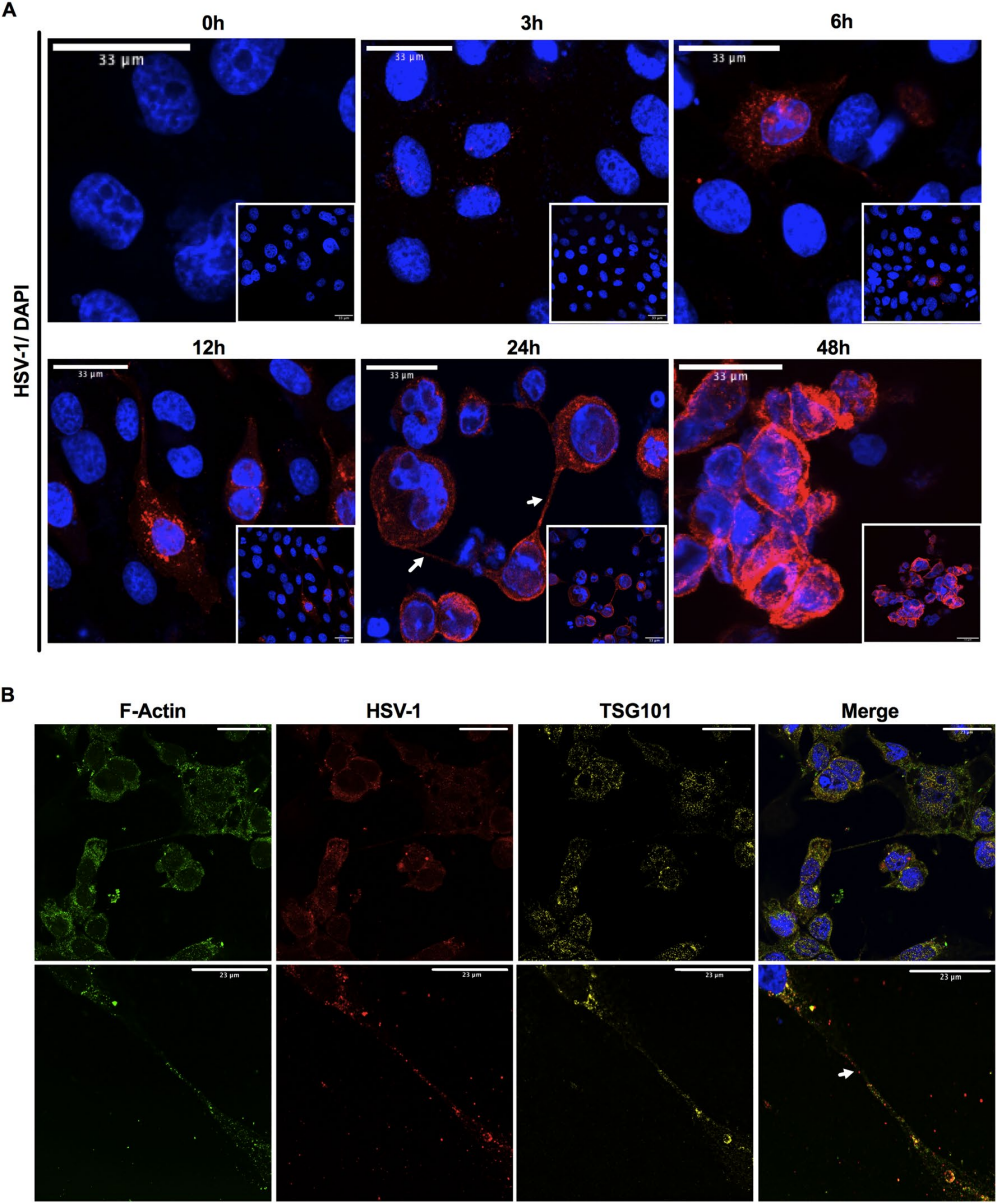
HSV-1 infected human corneal epithelial cells. (**A**) Progression of HSV-1 infection over 48 h, with virus visualized after staining with an anti-HSV-1 antibody (red) and DAPI (blue). Scale bars, 33 μm. (**B**) Infected HCECs stained with antibodies against F-actin (green), HSV-1 (red), TSG101 (yellow) and DAPI (blue), imaged on a Leica Stellaris STED super resolution microscope. Arrows indicate TNTs. Scale bars, 23 μm.

Immortalized human corneal epithelial cells (HCECs) that retained key features of primary cells^28^ were infected with an MOI of 0.5 of HSV-1 and followed over 48 h (Fig. 1A). At 3 h post-infection, only a few cells showed anti-HSV-1 antibody staining. The number of infected cells increased with time. By 12 h post-infection, cells had HSV-1 positive filopodia-like structures that by 24 h post-infection, had developed into TNT-like structures that were observed connecting multiple cells (Fig. 1A). By 48 h post-infection, almost all cells were positively stained. They were also rounded up and slightly detached from the tissue culture plate. The TNT-like structures were stained with F-actin, strongly supporting their identity as actin-rich TNT (Fig. 1B). Under a STED super-resolution microscope, an antibody against TSG101, an exosome marker, stained tiny spots within the TNTs and in the cytoplasm around the nucleus of infected cells (Fig. 1B). Anti-HSV-1-stained viruses were seen within the TNTs, but these were not co-localized with TSG101 positive staining. However, virus was seen in TSG101 positive structures outside TNTs in the cytoplasm.

### GF-19 peptide design

Several short LL37 derivatives were compared with the parent peptide (LL37) against a range of parameters in the design of a new anti-HSV-1 cHDP as shown in Table 1, with sequences in Supplementary Table S1. The parameters considered included net charge, Aliphatic Index and Instablity Index^29^. The hydrophobicity ratio^30^, Boman Index^31^, GRAVY Instability and Aliphatic Indices^32^ and predicted IC_50_ values^33^ were also evaluated. In general, antiviral peptides should be cationic and amphipathic in nature, allowing them to target and disrupt viral lipid envelopes and thereby preventing viral entry into the cells to block their replication^34^. We evaluated bioactive fragments of LL37 that had the cytotoxic N-terminal sequence removed. Among these, KR12, FK13, GF17 and GI20 have a net positive charge of 5, close to that of LL37. GF17 showed the highest hydrophobicity at 47%.

**Table 1:** Properties of the cathelicidin LL37 and its fragments as candidate anti-HSV-1 peptide. GF19 is the novel peptide designed for the present study. Sequences are shown in Supplementary Table S1.

Peptides	Net charge at pH 7	Hydrophobic ratio (%)	Boman index (Kcal/mol)	GRAVY index	Predicted in silico anti-viral IC50 (µM)	Instability Index	Aliphatic Index
LL37	+ 6	35%	2.99	− 0.724	36.6	23.34	89.46
KR12	+ 4	41%	4.02	− 0.708	63.06	31.95	121.67
FK13	+ 4	46%	3.48	− 0.438	3.85	18.70	112.31
GI20	+ 4	45%	2.47	− 0.225	0.64	13.34	126.50
GF17	+ 4	47%	2.47	− 0.094	1.43	23.91	125.88
GF19	+ 5	47.37%	2.24	− 0.089	1.21	16.46	133.16

The Boman Index estimates the potential for a protein to bind to other proteins^31^. The higher the index, the more likely the protein will have multiple roles within the cell. For anti-infective activity, a lower Boman index is preferable to minimize undesired side interactions^35^. GI20 and GF17 have the lowest Boman Index of 2.47 kcal/mol.

The number of hydrophobic residues within a HDP is proportional to its hemolytic and anti-infective activities, while the number of positively charged residues is proportional to antimicrobial activity and inversely proportional to hemolytic activity. Many cHDPs contain ≥ 50% hydrophobic residues^36^ but such residues could be very hemolytic. Positively charged residues could buffer the hemolytic effect of hydrophobic residues, and therefore, to minimize cytotoxicity, the hydrophilic/hydrophobic residue ratio of the new cHDP should be high to obtain the highest possible positive charge while maintaining a hydrophobicity of one-third or higher of the amino acid residues. The Aliphatic Index provides the relative volume of a peptide occupied by aliphatic side chains while the GRAVY (Grand average of hydropathicity) Index measures the hydrophobicity (positive) or hydrophilicity (negative), with both these indices being useful for predicting antiviral activity^32^. Previous studies showed that the Aliphatic Index for antiviral peptides effective against HSV-1/2 were between 19.33 to 146.79, with the GRAVY Index from − 1.121 to + 1.288^32,34^. The Instability Index measures the stability of a protein, with a value < 40 indicating a stable protein. LL37 and all its fragments fit into these ranges.

We had previously tested the efficacy of KR12, the smallest fragment and showed it was cell-friendly with significant in vitro but only mild antiviral activity in vivo in rabbits^34^. FK13 was another candidate that was eliminated as the exposed N-terminal phenylalanine suggested a high cytotoxicity level borne out in early in vitro tests (unpublished data). As the aim was to be able to load as much peptide as we could into our nanoparticulate delivery system, a smaller peptide was more desirable and GF17 was selected over GI20.

GF-17 (GFKRIVQRIKDFLRNLV) corresponds to residues 17–32 in LL37, with glycine (G) at the N-terminal, replacing glutamic acid (E) in the original LL37 sequence. Residue sequence number 18 (Lysine – K) and C-terminal residues 29–32 (arginine-asparagine-leucine and valine – RNLV) were reported to be critical for antiviral activity against Zika^25^. Addition of extra amino acids to the N-terminal of GF17 to form GI20 did not increase its antiviral activity against HIV^26^. Hence, we left the N-terminus unmodified.

We modified the C-terminus with 2 additional amino acids, lysine (K) and leucine (L), using an amidated carboxyl group (-CONH_2_) to further enhance the efficacy of peptide in penetrating viral membranes. We predicted that the addition of -KL at C-terminus would not affect the helical structure of peptide sequence and will improve its efficacy. Lysine, with a pK of 10.5 in its side chain group, was added to provide the positive charge in the peptide sequence. Leucine was added as a hydrophobic amino acid known for rupturing membranes^37,38^.

The prediction of peptide antiviral activity in terms of half maximal inhibitory concentration (IC_50_) is a very useful measure in designing new peptides. The antiviral peptide (AVP)-IC_50_Pred is a prediction model developed to predict IC_50_ values^34^. The server at http://crdd.osdd.net/servers/ic50avp was used and gave a predicted IC_50_ of 1.21 for GF19. The predicted characteristics of GF19 are shown in Table 1.

### In silico testing of GF19

**Figure 2:**
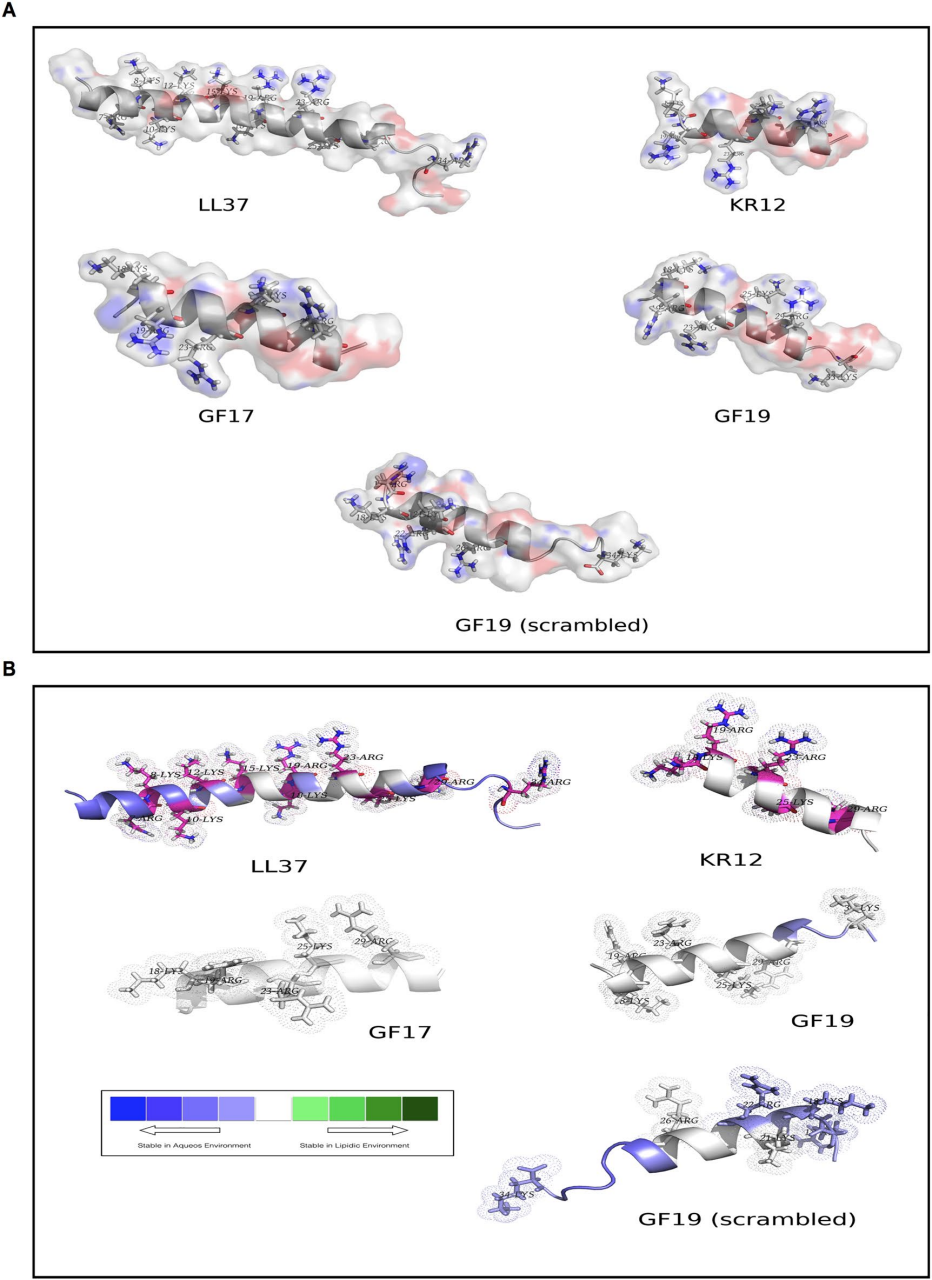
PyMol^40^ depictions of GF19 compared to the LL37 parent molecule and its other fragments. (**A**) The designed, full-length peptide structures with their accessible surface area (ASA) are shown with a coloring scheme based on atom types (N: blue, O: red). Positively charged residues (arginines, lysines) are presented as sticks with their van der Waals surfaces presented as semi-transparent solid surfaces to render the solvent accessible areas. (**B**) BRANEart display of the peptides colored according to the Membrane Propensity index (MPr). Comparative stability of the peptides in an aqueous solution / environment can be interpreted in terms of the BRANEart colourbar where increasing blue intensity represents increasing stability in an aqueous environment, while increasing green intensity represents increasing instability in the same environment. This, in turn, can be interpreted in terms of less and more reaction proneness respectively for the two cases, following an inverse correlation. Positively charged residues (arginines, lysines) are presented as sticks with their van der Waals surfaces presented as dots.

The in silico reactivity of GF19 compared to GF17, the parent LL37 peptide and the bioactive KR12 core fragment present in all the peptides were compared. We also examined a scrambled version of the GF19 peptide that was used as a control for GF19 activity (GF19_scrambled_ or sGF19). The accessibility score (*rGb*)^39^ is a direct measure of stability (chemical inertness) of a protein or peptide in an aqueous solution, which can further be extended to explain its chemical reaction proneness (i.e., aqueous instability). A threshold *rGb* value of 0.011 or less suggests that the peptide species is unstable and reactive. Natively folded globular proteins as well as protein–protein complexes entrapped in an aqueous environment had an average *rGb* of ~ 0.057 (SD: 0.022)^39^. As can be seen in Fig. 2, all (designed, full-length) peptides were unstable (thus, reactive) in an aqueous environment (with negative *rGb* scores), while *rGb* of all designed peptides have magnitudes ~ 4–5 times higher than that of LL37. This suggests that all designed peptides (including GF19_scrambled_) were more reactive standalone molecular species than LL37. The reaction-proneness is likely due to their significantly smaller sizes (from ~ one fourth to half the length of the full-peptide) compared to that of LL37. While the full-length helical peptide has a bend in its main-chain trajectory (due to charge and steric repulsion of closely clustered arginines and lysines), the designed smaller-sized peptides were much more linear in their backbone trajectories, making their hydrophobic residues / patches more exposed to the (aqueous) solvent, thereby leading to the greater instability and reaction proneness (Fig. 2A).

A second measure, the Membrane Propensity index (or MPr score)^40^, was implemented as a cross-validation method in a complementary approach to that of *rGb*. MPr describes the membrane preference of a protein unit which can, in turn, be redirected to indirectly interpret the aqueous preference of the globular protein units. The higher the MPr score, the higher is the preference (stability) for the concerned protein unit for lipidic (or, hydrophobic) environments. Alternatively, the lower the MPr score, the higher is its preference (stability) in an aqueous environment. KR12, the smallest active fragment of LL37 with weak anti-HSV-1 activity^23^, GF17 and GF19 all had ~ 1.5 times higher average MPr scores (MPr, averaged over all residues pertaining to the corresponding peptides) compared to that of LL37, which further suggests that all designed peptides (except GF19_scrambled_) are more vulnerable (or energetically frustrated) in an aqueous solution than LL37 and are therefore more reaction-prone. GF17 and KR12 had similar degrees of instability, while GF19 was slightly less unstable in an aqueous environment. Distribution of the inhomogeneous instabilities locally on the peptide structures could be seen from the comparative visual portray of the BRANEart^40^ MPr map on LL37 and the designed peptide structures (Fig. 2B). The local distribution of the aqueous instability could also be revealed by a greater extent of neutral (white) preference zones (which are not so stable in water) in all designed peptides compared to LL37. The overall results from both *rGb* and MPr modeling measurements suggest that the judicious design of the modified peptides could improve the reaction proneness of the original full-length peptide. Interestingly, GF19_scrambled_ had an MPr score similar to LL37 (with roughly equal and low standard deviations) rather than the rest of the designed peptides. From the local distribution (Fig. 2) also, the two profiles (LL37, GF19_scrambled_) resembled each-other by having a much greater extent of bluish patches than the rest of the designed peptides. Scrambled GF19 had the identical amino acid composition to that of GF19 with its sequence scrambled, designed to check for any positional bias of the amino acid residue positions in GF19 that might be responsible for its reaction proneness (or, aqueous instability) and biological function. The MPr results showed that the exact sequence of GF19 was required to render its characteristic effective aqueous instability, which could not be replaced by the scrambled sequence with an identical amino acid composition.

### Biocompatibility and immune compatibility of GF19

**Figure 3:**
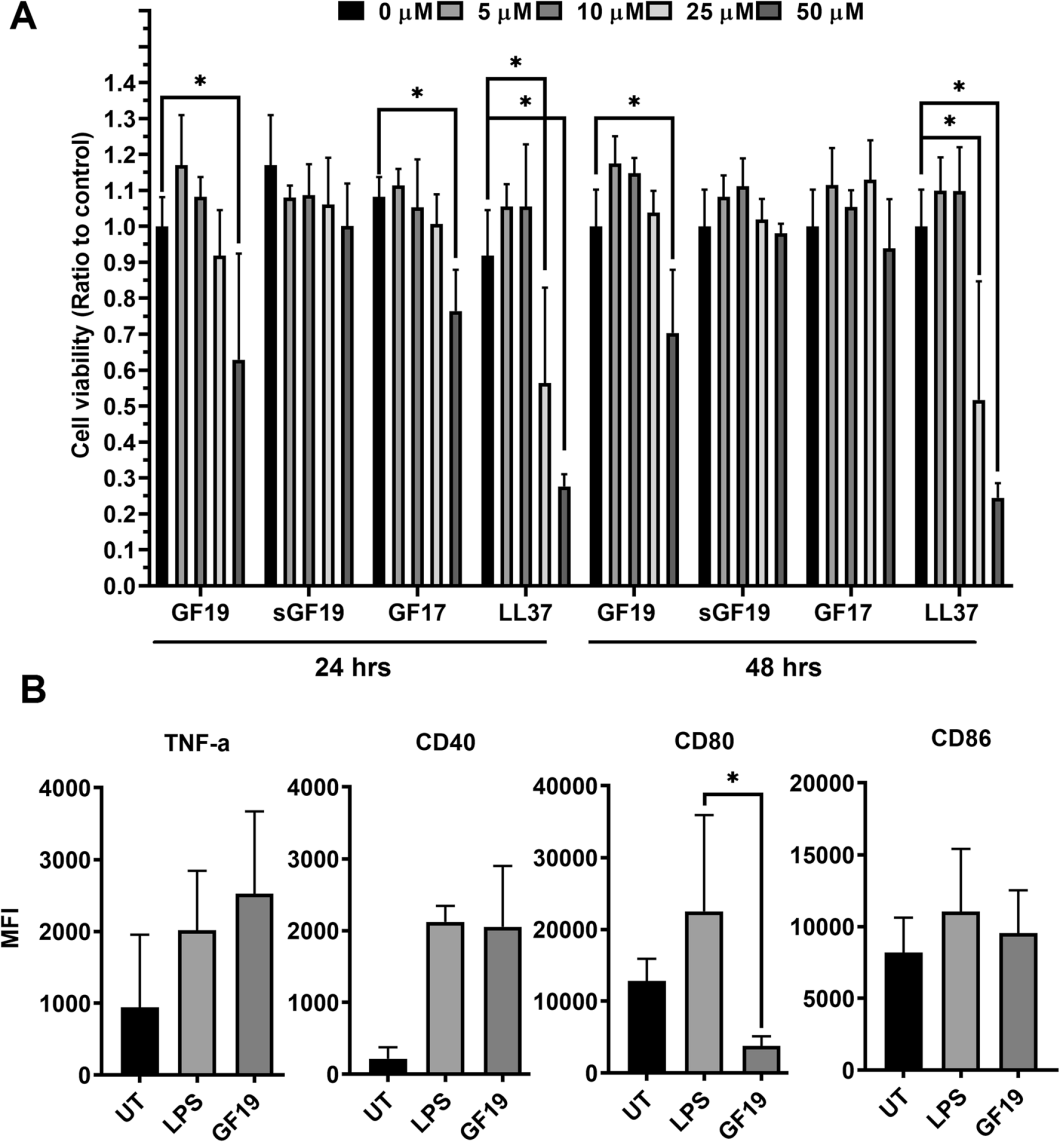
Biocompatibility and immune compatibility of antiviral peptides. (**A**) Effect of antiviral peptides GF19, its scrambled counterpart (sGF19), GF17 and LL37 at 24 and 48 h post-treatment at concentrations of 5, 10, 25 and 50 µM on the cell viability of HCECs; n = 6 samples per group. (**B**) Effect of GF19 treatment on BMDC activation, compared to that of LPS (positive control). Mean fluorescence intensity (MFI) of TNF-α, CD40, CD80, and CD86 from untreated (UT), GF19-treated and LPS-treated BMDCs are presented. Asterisks indicate significance compared to positive control, LPS, after analysis with a one-way ANOVA and post-hoc Dunnett’s test. Significance was set at P ≤ 0.05. Error bar indicates SD.

Cultured HCECs were used to determine the cytotoxicity of the LL37, GF17, GF19, and scrambled GF19 (sGF19) peptides (Fig. 3A, Supplementary Tables S3, S4). AlamarBlue™ was used to track HCEC proliferation in the presence of different concentrations of peptides. Control samples were cells incubated in culture media only (0 µM). Cell viability was expressed as a ratio of their numbers at 24 or 48 h of culture compared to the control (Fig. 3A). The results show that from 5 to 25 µM, only LL37 showed significant cytotoxicity at 25 µM, with observed average cytotoxicity of 48% at 24 h and 49% at 48 h. At 50 µM, all the peptides except sGF19 showed significant decreases in the cell viability (48% for GF19, 32% for GF17 and 73% for LL37, at 24 h). At 48 h, 50 µM of GF19 and LL37 showed significant cytotoxicity, while only LL37 was cytotoxic at 25 µM.

To test the immunogenicity of GF19, we cultured primary bone marrow-derived dendritic cells (BMDCs) with GF19 or lipopolysaccharide (LPS), the latter, a bacterial toxin, as positive control. GF19 induced the expression of TNF-α and CD40 on BMDCs to levels comparable to that observed with LPS (Fig. 3B). This suggests that GF19 induces an inflammatory response, at least in BMDCs. However, while LPS induced a modest increase in CD80 and CD86 expression, GF19 did not induce CD86 expression, and CD80 expression was decreased relative to control (Fig. 3B). As such, CD80 expression was significantly lower in GF19-treated cells compared to LPS. Altogether, these data suggest that GF19 induces an inflammatory response in BMDCs that does not lead to an increase in costimulatory ligands CD80 and CD86, which are required to activate naïve T cells. Therefore, GF19 may create an inflammatory environment, while bearing tolerogenic properties with regards to the adaptive immune response.

### Antiviral efficacy of GF19 against HSV-1

**Figure 4:**
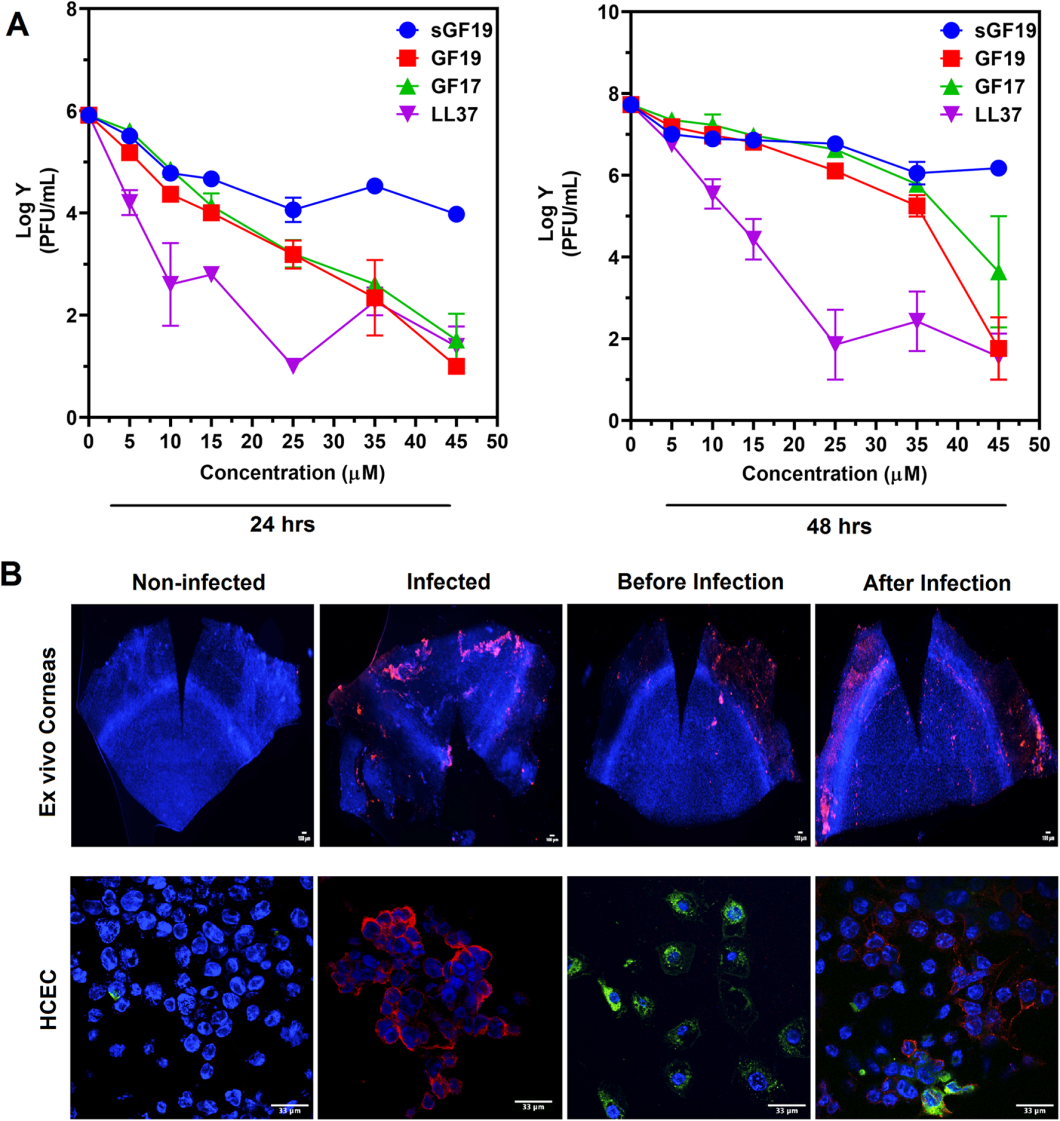
Antiviral activity of GF19. (**A**) Antiviral activity GF19 compared to a scrambled control, sGF19, and the peptides it was derived from, GF17 and LL37 as shown by plaque assay counts at 24 h and 48 h after treatment. Concentrations of peptides from 0 to 45 µM were examined. Data are significant compared to the positive control, LPS, after analysis with a two-way ANOVA and post-hoc Tukey test, with P ≤ 0.05 (Table S5). Error bar indicates SEM. (**B**) Infected ex vivo mouse corneas and human corneal epithelial cells (HCEC) treated with GF19. GF19 was given either before infection (before) or immediately after infection (after); HSV-1 stained with AF594 (red), FITC-GF19 (green), DAPI (blue). Scale bars, 33 µm.

The antiviral efficacies of GF19 and its associated peptides were measured by exposing HCECs to MOI 1 of HSV-1 and the various peptides for one hour. The curves in Fig. 4A that were generated by the effects of the plaque counts resulting from the ability of the different peptides to block virus replication within the HCECs show the differences in efficacy of the peptides in blocking viral replication. The steeper the slope, the more efficacious the peptide was at stopping HSV-1 activity.

LL37 was the most effective at blocking HSV-1 replication, followed by GF19 and then GF17 (Fig. 4A). The scrambled GF19 (sGF19) peptide was the least effective at blocking viral replication even though it contained the identical amino acid content as GF19, confirming our in silico modelling results that the exact GF19 sequence was needed for its antiviral activity.

Statistical analysis (Supplementary Tables S5, S6) showed that after only 24 h, there was a significant difference between GF19 at concentration of 10 μM and control, while such a significance was not detected in GF17. At 48 h post-treatment, although GF19 still showed higher amounts of reduction in number of plaques, both GF19 and GF17 showed significant antiviral activity at 35  μM and 45  μM of peptide. Nevertheless, GF19 was more efficacious than GF17, with more potent antiviral activity over a shorter time.

To further understand the antiviral activity of GF19, infections were performed on ex vivo mouse corneas in organ culture. Each cornea was treated with 45  μM GF19. “Before treatment” refers to the corneas being given GF19 one hour before infection with virus, while “after treatment” refers to GF19 given immediately after HSV-1 infection. In both cases, the corneas were exposed to virus for one hour after which media was removed and fresh media with only GF19 treatment was added for 24 h post-infection. We found that the HSV-1 virus was localized to the limbal region rather than the central cornea in both treated and untreated ex vivo tissue. HSV-1 staining was more intense in the infected and untreated tissue, than the corneas given GF19 treatment after infection. Conversely, the ex vivo corneal tissue pre-treated with GF19 showed less HSV-1 antibody staining.

The immunocytochemistry results of the monolayer cultures of HCECs in Fig. 4B showed that almost all the cells were infected by HSV-1 when left untreated over 24 h. The cells that were pre-treated with FITC-GF19 were labelled green, showing that the peptides were taken in by the cells. The staining was in discrete green spots suggesting the peptide could have been sequestered in vesicular structures within the cells. There was minimal HSV-1 staining. For treatments given after infection, however, there were markedly more anti-HSV-1 stained cells. There were very few cells that had taken up the FITC-GF19. These results showed that GF19 was more effective when given to the cells prior to HSV-1 infection, most likely blocking viral entry into the cells.

### Encapsulation of GF19 in silica NPs

**Figure 5:**
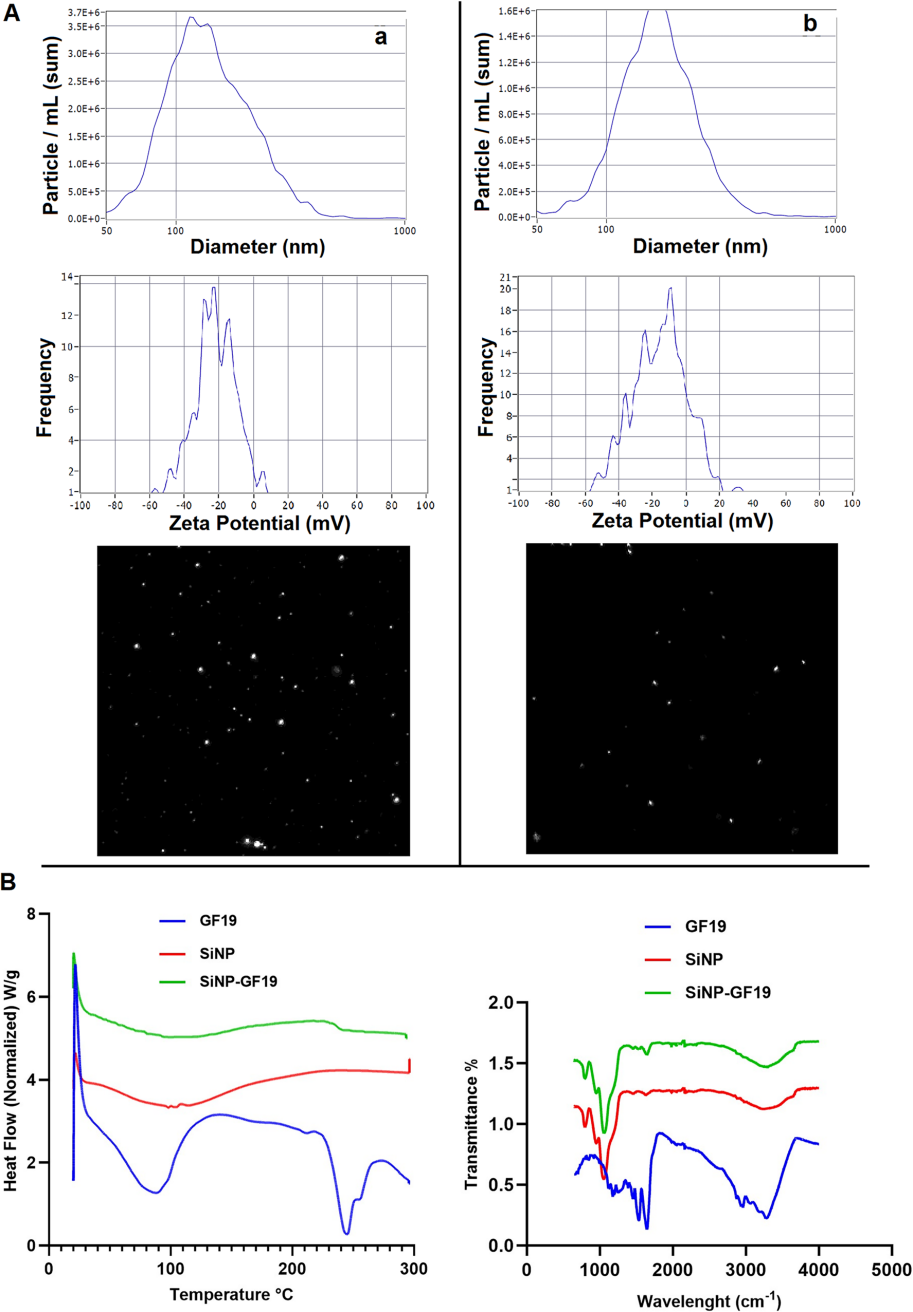
Characterization of SiNPs with and without GF19. A) NTA-ZetaView analysis showing the size (nm) and zeta potential (mV) of) SiNPs only (**a**) and SiNP-GF19 (**b**). B) DSC curves and FTIR spectrum of GF19, SiNPs, and SiNP-GF19.

The diameters of SiNPs and SiNP-GF19 were 131.5 ± 69.00 nm and 162.20 ± 71.30 nm, respectively, and encapsulation efficiency (EE) was 97.0 ± 1.20%. The successful incorporation of GF19 into SiNP was confirmed by the increased particle size of SiNP-and change in zeta potential from − 50.89 ± 0.43 mV to − 47.19 ± 0.54 mV (Fig. 5A). A differential scanning calorimetry (DSC) study performed (Fig. 5B) showed the presence of a single broad endothermic peak at around 100 °C for SiNP which can be attributed to the evaporation of physically absorbed water^41^. GF19 exhibited two distinct endothermic peaks observed at 90 °C and ~ 240 °C that are likely associated with the melting point of the peptide, while SiNP-GF19 showed a much-diminished endothermic peak ~ 240 ℃. The DSC thermogram of SiNP-GF19 showed a noticeable decrease in the intensity of the majority of GF19 peaks. The lack of a melting peak observed in the DSC curve of GF19 can be attributed to the formation of an inclusion complex between the drug molecules and SiNPs. The absence of distinct DSC peaks corresponding to the melting point of GF19 indicates that the peptide molecules were effectively encapsulated and were present in an amorphous or semi-crystalline state within the pores. The FTIR analysis of SiNPs revealed distinct absorption peaks at wavelengths of 1060 cm^−1^, 797 cm^−1^, and 3396 cm^−1^. These peaks can be attributed to the bending motion of Si–O-Si and the stretching vibrations of Si–O (siloxane) and Si–OH (silanol), respectively^42^. The GF19 spectrum has prominent peaks at approximately 1600 cm^−1^, which have been attributed to the vibrational modes associated with the N–H and N–C bonds. The presence of the peak at approximately 3000 cm^−1^ has been verified to be associated with the amine groups present in lysine. The incorporation of GF19 into SiNPs resulted in a significant suppression of nearly all peaks associated with GF19, except for a small peak observed at 1456 cm^−1^, which suggests the presence of GF19 in SiNP-GF19 (Fig. 5B).

### GF19 interaction with human corneal epithelial cells

**Figure 6:**
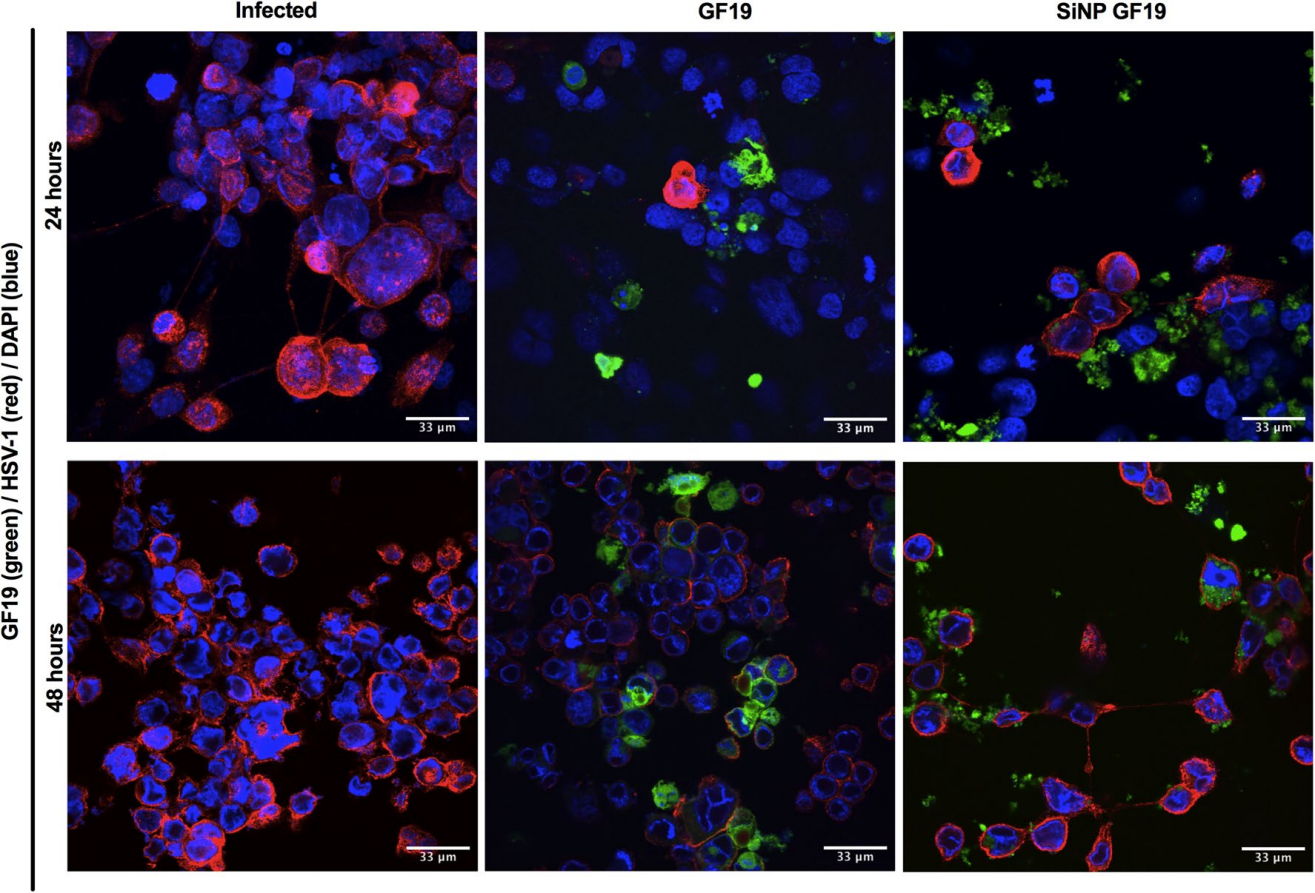
HSV-1 infected human corneal epithelial cells treated with GF19 or silica nanoparticle encapsulated GF19 (SiNP-GF19) and observed after 48 h. The cells were stained with an antibody against HSV-1 (red) and DAPI; GF19 was tagged with FITC. Scale bars, 33 µm.

HCECs were infected with MOI 0.5 and treated with a low concentration of 2.5 µM of either GF19 only or silica nanoparticle encapsulated GF19 (SiNP-GF19), where the GF19 was tagged with FITC. All cells were pre-treated with GF19 or SiNP-GF19 for 1 h prior to viral infection. Media was removed after one hour and fresh media without virus or treatments was added. At 24 h after infection, large, rounded cells stained red with the anti-HSV-1 antibody were seen in the infected untreated cultures. The rounding of cells is typical of the cytopathic effects described for herpes virus infections^43^. At 48 h, infected but untreated cells formed clumps that were detaching from the dishes. HCECs treated with free GF19 and SiNP-GF19 showed labelled peptide localized to cells without HSV-1 staining at 24 h post-infection, suggesting that the GF19 blocked viral entry. At 48 h, most cells are surrounded by red fluorescent anti-HSV-1 staining. The stained cells were also rounded. However, a proportion of SiNP-GF19-treated cells remained attached to the substrate with intact TNT formation even at 48 h compared to cells treated with free GF19 or untreated cells (Fig. 6). These results suggest that GF19 was slowing down the pace of infection in corneal epithelial cells, most likely by blocking viral entry.

## Discussion

We show that HSV-1 in cultured human corneal cells spread to neighbouring cells through TNTs as described for other viruses. TSG101 labelled vesicular structures were observed under super-resolution but these did not co-localize with virus particles within the TNTs. However, TSG101 vesicle-like structures colocalized with virus particles within the cytosol of infected cells, showing that exosomes were also involved in viral spread. TNTs seem to be the more important cell-communication system during viral infections, though the interplay between exosomes and TNTs is still not understood.

Corneal HSV-1 infections are often treated systemically with Acyclovir (ACV) or one of its associated prodrug or derivatives, to control viral infection as drug penetration through eye drops is very poor^2^. Once infected, HSV-1 undergoes latency in the trigeminal ganglia and possibly in the cornea, where it can reactivate. Reactivation as opposed to the primary infection, is associated with intense inflammation and keratitis (HSK). ACV and ganciclovir have been prescribed as prophylactics but there are multiple reports of reoccurrence and resistance^10,44^ as well as poor uptake into corneal tissues^45^. The cationic peptide LL37 and more extensively, its bioactive fragments with the cytotoxic N-terminal sequence (residues 1–12)^46^ removed, have been explored as an alternative treatment for HSV-1 infections. From these shorter segments with reduced cytotoxicity, we designed and modelled GF19, a modified cHDP based on a series of design criteria for antimicrobial peptides in general but also taking into consideration the differences between HDPs with antibacterial vs antiviral efficacy. In a study of 604 antiviral peptides, Chang and Yang^32^ examined the properties that conferred antiviral efficacy. They reported that effective antiviral peptides tended to contain more aliphatic residues (leucine, isoleucine, and valine), had average instability values around 40 (i.e., relatively unstable), with high positive charge. The majority of the efficacious antiviral peptides were alpha helices, and many contained lysine (K) residues. Our GF19 contained the properties of the GF17 peptide with documented antiviral activity and added on another lysine and leucine to further enhance efficacy.

Hydrophobic amino acids such as leucine hold the ability to disrupt lipid bilayer membranes^37,38^, while lysine is highly positively charged. Together, addition of -KL to the -C terminal of GF-17 theoretically enhances the membrane rupturing ability of GF19. We showed in silico and confirmed experimentally that GF19 with its additional -KL peptides were more reactive and had higher antiviral activity than GF17.

The antiviral activity of GF19 was not as efficient as LL37 but it was not cytotoxic like LL37 either. The in silico prediction that the exact sequence of GF19 was necessary for its antiviral activity was borne out by the actual poor antiviral activity of the scrambled GF19 peptide in the HCEC cultures.

We showed in Lee et al.^21^ that LL37 was not able to clear HSV-1 from infected corneal cells but was able to slow down viral spread for up to 72 h. GF19 when used to pre-treat cells and ex vivo mouse corneas, was able to block viral entry into cells effectively. When GF19 was given immediately after exposure to HSV-1, it was not able to clear the infection but slowed down the spread. These results suggest that GF19 actions are more suited for its use as a prophylactic then a virucidal drug. The next step would be to investigate the efficacy of the peptide as a prophylactic, such as in preventing reactivation of latent virus during corneal surgery, or to prevent transmission of virus from possibly infected donor tissue. The use of GF19 to block viral entry in combination with an antiviral drug like ACV that works on preventing viral replication once the virions are internalized will also merit testing.

## Materials and methods

### Viral stock and plaque assays

The HSV-1 McKrae strain was used for this study (a gift from Dr. Daniel J. Carr, Univ. of Oklahoma Heath Sciences Centre, Oklahoma, USA). The virus was propagated in Vero cells (ATCC, CCL-81) using DMEM-Hi glucose (Sigma-Aldrich, St. Louis, MO, USA) supplemented with 10% fetal bovine serum (FBS; Wisent, St-Bruno, QC, Canada) and penicillin–streptomycin (Gibco™, Thermo Fisher Scientific, Waltham, MA, USA). Virus was collected after 3 days of infection, mixed with an equal volume of milk and freeze-thawed four times using dry ice. Stocks were maintained at −80 °C until further usage. Viral titers were obtained by performing plaque assays.

For plaque assays, Vero cells were grown in either 6- or 12-well plates in DMEM-Hi glucose supplemented with 10% FBS and penicillin–streptomycin. Once the cells were 90–95% confluent, media was removed and serum-free media containing the virus at varying concentrations was added. Cells were incubated with the virus for one hour, with shaking every 15 min. The viral suspension was removed, and the cells were rinsed with 0.01 M PBS. A liquid overlay consisting of 1.2% Avicel®PH-101 (registered trademark of FMC Corp; Supelco distributed by Sigma-Aldrich, St. Louis, MO, USA) in DMEM-Hi glucose^47^ was applied. After 3 days, the liquid overlay was carefully removed, and cells were fixed with 10% formaldehyde for 30 min. 0.5% crystal violet was used to stain the cells and count the plaques.

### HSV-1 infection of HCECs

Immortalized HCECs^28^ (from Dr. H. Handa, Division of Ophthalmology, Kinki Central Hospital, Hyogo, Japan) were grown overnight on 12-well chambered slides (Ibidi GmbH, Gräfelfing, Germany) to 80% confluence, in KeratinoMAX medium (Wisent, St-Bruno, QC, Canada). The cells were infected with HSV-1 McKrae strain with an MOI of 0.5 for one hour, after which the virus-containing media was removed. Fresh media was added, and cells cultured for an additional 0, 3, 6, 12, 24 and 48 h, after which they were fixed in 4% paraformaldehyde for immunocytochemistry (n = 2 separate experiments with 3 replicates per time point). A polyclonal anti-HSV-1 virus antibody raised in goat (FisherSci PA17493, ThermoFisher, USA) and a donkey anti-goat IgG secondary antibody conjugated with DyLight™ 550, which gives an orange-red fluorescent signal, were used to detect virus-infected cells. Antibodies for F-actin staining of the microfilaments forming TNTs, and TSG101 exosome markers were used (Table 2). Imaging was performed on Zeiss Confocal LS M880 upright multiphoton system and Leica Stellaris 8 (confocal and STED modules). Image Analysis was performed using Fiji and Adobe Photoshop software.

**Table 2:** Antibodies used for immunofluorescence and flow cytometry.

Target	Antibody	Dilution Factor
HSV-1	HSV Type 1 Goat anti-Virus, Polyclonal, Invitrogen, PIPA17493	1:100
TSG101	Anti-TSG101 antibody [EPR7130(B), Abcam, ab125011	1:100
F-actin	F-actin Monoclonal Antibody (NH3), Invitrogen, MA180163	1:100
Mouse IgG	Goat anti-Mouse IgG (H + L) Highly Cross-Adsorbed Secondary Antibody, Alexa Fluor™ Plus 647, Invitrogen, A32728	1:1000
Goat IgG	IgG (H + L) Cross-Adsorbed Donkey anti-Goat, DyLight™ 550, Invitrogen, PISA510087	1:1000
Rabbit IgG	Goat anti-Rabbit IgG (H + L) Highly Cross-Adsorbed Secondary Antibody, Alexa Fluor™ 488, Invitrogen, A11034	1:1000
TNF-α	PerCP/Cyanine5.5 anti-mouse TNF-α, (Clone: MP6-XT22), (IstoType: Rat IgG1, κ), (Reactivity: Mouse), (Format: PerCP/Cyanine5.5), (APP: FC), (Species: Rat), BioLegend®, 506,321	1:800
CD40	CD40, APC, clone: 1C10, eBioscience™, 501,129,392	1:400
CD80	PE anti-mouse CD80, (Clone: 16-10A1), (IsoType: Armenian Hamster IgG), (Reactivity: Mouse, Cross-Reactivity: Dog (Canine)), (Format: PE), (APP: FC), (Species: Hamster), BioLegend®, 104,708	1:1600
CD86	FITC anti-mouse CD86, (Clone: GL-1), (IsoType: Rat IgG2a, κ), (Reactivity: Mouse), (Format: FITC), (APP: FC), (Species: Rat), BioLegend®, 105,006	1:50

### Modeling and in silico testing of designed peptide 3D structures

The atomic coordinates of human LL37 were extracted directly from the corresponding NMR structure of the antimicrobial peptide (PDB ID: 2K6O). Since KR12 was a direct subset of the full peptide (with no amino acid substitutions), its coordinates comprising residues 18–32 was extracted from the LL37 experimental structure. For designed peptides with amino acid substitution(s), (namely, GF17, GF19 and the scrambled baseline: GF19_scrambled_), MUSCLE^48^ was used to align the designed sequences pairwise (as targets) against the amino acid sequence of the full peptide, LL37. Then, these alignments were used to model the peptide structures using the ‘automodel’ module of MODELLER^49^ version 10.1. Carboxylic group of the C-terminus of the peptide was converted to an amide to mask the negative charge. Amidation was performed by a 2-step process, in the first step, the terminal most oxygen atom (OXT) of the C-terminal carboxylic groups were altered to nitrogen and, in the second step the hydrogens were added to them by ‘third and fourth atom fixation’ techniques^39,50^. All designed peptide structures, thus obtained, were then taken for short energy minimization in Gromacs v2021.3^51^ using its steepest descent algorithm until convergence (~ 100 steps) with a maximum number of steps set to 250. This ensured removal of any conformational bias in the designed peptides that might have occurred due to direct extraction of coordinate subsets (e.g., in the case of KR12) from the NMR structure of the full peptide, LL37.

#### The accessibility (rGB) score

As previously formulated and successfully used in several earlier publications^39,52–54^, the accessibility score (*rGb*) computes and compares the hydrophobic burial profile of a globular protein unit (proteins, peptides, protein complexes, protein domains) with respect to their general trends (or corresponding native distributions), enumerated from standard databases. The function measures the distribution of amino acid residues in a folded protein (or peptide) chain (or associated chains) as a function of solvent exposure and returns its compatibility fitness (or the extent of stability) in an aqueous environment. It is an integral part of the Complementarity Plot^39,55^. The score is analytically designed based on normalized conditional probability (or propensity) estimates of residue types (e.g., Val, Asn, His, etc.) given their burial (and hence the name: *rGb*: *residue Given burial*) and can be formulated as follows.

$$rGb=\frac{1}{{N}_{res}}{\sum }_{i=1}^{{N}_{res}}{log}_{10}\left({Pr}_{i}\right)$$

where *N*_*res*_ is the sequence length of the input polypeptide chain and *Pr*_*i*_ is the propensity of a particular amino acid (Val, Asn, His etc.) to acquire a particular degree of exposure to aqueous solvent.

A value of *rGb* > 0.011^6^ (and higher the better) renders the input atomic model affirmative with regard to the ‘native-like’ distribution of amino acids in terms of their hydrophobic burial, while a value less than that means hydrophobic residues are exposed to the solvent causing the molecule stay in an unfavorable / frustrated disordered (high entropy) state. A negative value emphasizes that instability can be severe and can be used to signal the reaction-prone nature of the said protein (or peptide) unit. Thus, by definition, *rGb* serves as a measure of stability / meta-stability / reactivity of a protein unit in aqueous environment.

Prior to computing the *rGb* score, the solvent accessible surface area (ASA) were computed by NACCESS^56^ following the standard Lee and Richards protocol^57^ and burial (bur) of ASA for each amino acid residue pertaining to the peptide(s) were computed following standard procedure^58^.

#### The membrane propensity index (MPr score)

The MPr score^40^ describes the membrane (or lipidic) preference of a protein unit under test and is primarily designed to serve to identify stability strength and stability weaknesses in the trans- and extra-membrane regions of membrane proteins. However, as already demonstrated earlier^40,54^, its applications are not limited to membrane proteins but rather it can be used to indirectly interpret the aqueous preference of the globular protein units as well. The higher the MPr score, the higher the preference (stability) for the concerned protein unit for lipid environments, while lower the MPr score, higher is its preference (stability) in an aqueous environment. MPr scores for the concerned peptides were directly obtained from the BRANEart web-interface (http://babylone.3bio.ulb.ac.be/BRANEart/index.php) along with meaningful visual representations of the structures colored according to the stability index, MPr.

### Biocompatibility

HCECs from an immortalized line that retain the key characteristics of primary cells^28^ was used to determine the biocompatibility of GF19. GF19, and controls, namely scrambled GF19, GF17 and LL37, and FITC-labelled GF19 were custom synthesized by Advanced Peptide Synthesis Services, Beats Research (https://www.beatsresearch.com/aps.php, Ottawa, Canada). To determine the biocompatibility of the various peptides, HCECs were seeded in 96 well plates containing KeratinoMAX medium (Wisent, St-Bruno, QC, Canada) with human recombinant epidermal growth factor (rEGF) and bovine pituitary extract (BPE) as supplements. An initial IC50 test done to obtain the concentration where 50% of cells are killed. Together with previous studies of the bioactive KR12 region^23^ within GF19 and the full-length LL37 parent peptide^21^, these early results helped to determine the test concentrations to be tested. HCECs were incubated with varying concentration of the peptides (0, 5, 10, 25 and 50 µM) for 24 h. Six samples were analyzed for each concentration. After the appropriate incubation, the cell proliferation was analysed using colorimetric alamarBlue™ (Invitrogen, Thermo Fisher Scientific, Eugene, OR, USA) cell viability assay. Ten µL of alamarBlue™ dye was added in each well, incubated for 3 h at 37 °C in a humidified 5% CO2 incubator and colorimetric analysis was done by measuring absorbance at 570 nm, according to manufacturer’s protocol.

### Immunogenicity: BMDC culture and flow cytometric analysis

After ethical permission from the Animal Care and Use Committee of Maisonneuve-Rosemont Hospital (protocol #2023 - 3239) and in accordance with euthanasia guidelines from ARRIVE guidelines and the Canadian Council of Animal Care, five C57BL/6 J mice (6 to 12 weeks old males) were euthanized. The tibias and femurs were dissected out and bone marrow was obtained for further in vitro studies. In a 24-well plate, 10^6^ bone marrow cells per well were cultured in 2 mL of RPMI 1640 containing 10% (v/v) fetal bovine serum (FBS) (Wisent, St. Bruno, QC, Canada), penicillin–streptomycin-glutamine (0.5 mg/mL), 10 mM Hepes, 1 mM sodium pyruvate, 55 μm of β-mercaptoethanol, and granulocyte–macrophage colony-stimulating factor (2.5 ng/mL; GM-CSF) (all from Gibco™, Thermo Fisher Scientific, Waltham, MA, USA). After 2 and 3 days from the initial seeding, 1 mL of media was replaced with fresh media containing 5.0 ng/mL of GM-CSF. On day 6, the cells were collected and Histodenz™ (Sigma-Aldrich, St. Louis, MO, USA) was added to create a density gradient to isolate large cells. 10^6^ large cells were replated in 2 mL of media for treatment, with either 45 µM GF19, 1 µg/mL lipopolysaccharide (LPS) or were untreated. Once the treatment was added, cells were cultured for an additional 24 h. At 18 h, BD GolgiStop™ (Becton, Dickinson and Company, Franklin Lakes, NJ, USA) was added to prevent the secretion of cytokines. Cells were stained for surface markers CD11c, CD40, CD80, and CD86 (Table 2) and Zombie Aqua™, an amine-reactive green fluoresceny viability dye for flow cytometry (BioLegend®, San Diego, CA). Fixation and permeabilization was performed to allow for intracellular staining of TNF-α (Table 2). A BD LSR II flow cytometer was used on all the samples and were analyzed using FlowJo™ software (Becton, Dickinson and Company, Franklin Lakes, NJ, USA). All cell suspensions were sampled for the same duration and speed on the LSR II. BMDCs were selected as CD11c^hi^ viable cells. Mean fluorescence intensity (MFI) of TNF-α, CD40, CD80, and CD86 were compared among all samples and plotted as a graph. Each mouse was an experimental unit, n = 5.

### In vitro antiviral efficacy

HCECs were cultured in 48-well plates on top of 8 mm diameter cover slips in a humidified incubator at 37 °C and 5% CO_2_. The growth media used was KeratinoMAX serum-free medium with supplements (Wisent, St-Bruno, QC, Canada). Once cells were 90–95% confluent, the media was removed and fresh media containing the treatment and an MOI 1 of HSV-1 was added. Treatments included the antiviral peptides LL37, GF17, GF19 and scrambled GF19 (sGF19) at a concentration of 5, 10, 15, 25, 35 and 45 μM. Three replicates at each concentration were examined. Controls consisted of infected, non-treated cells and non-infected, non-treated cells (blank). Cells were incubated for an hour, rinsed with 0.01 M PBS, and given fresh media containing the treatment. After 24 h from the initial infection, the media was collected and stored at − 80 °C for plaque assays. Fresh media, not containing the treatment, was added to the HCE cells and after an additional 24 h, the media was collected and stored at − 80 °C. Cells were fixed with 4% PFA in 0.1 M TBS overnight at 4 °C. Plaque assays were performed as described above for titering.

### Treatments with GF19 before and after HSV-1 infection

#### Ex vivo corneas

Eyes from 12 C57BL/6 J mice (6 to 12 weeks old) were removed and rinsed with 0.01 M PBS containing 3 × penicillin–streptomycin. All corneas were scratched using a 2 mm diameter biopsy punch and were placed randomly into a 96-well plate. Two treatments with three different conditions were tested. Free GF19 and GF19 encapsulated in SiNPs were both tested at a concentration of 45 µM. For pre-treatments with GF19 infected eyes were either given media containing the treatment for an hour before the infection, or treated immediately following the initial infection. Infection only and non-infected, non-treated samples were used as controls. Infections were performed by diluting the virus to 10^4^ PFU in 200µL of DMEM-Hi glucose and submerging the eyes in it for 1 h with shaking every 15 min. Media for all samples were collected at 24 and 48 h for viral titering. For all eyes, after 48 h from the initial infection, samples were fixed with 4% PFA in 0.1 M TBS, overnight at 4 °C. After fixation, eyes were rinsed in 0.1 M TBS and used for flat mount staining.

For flat-mounts, eyes were dissected to consist of the cornea and a scleral rim. The corneas were stored in 0.1 M TBS at 4 °C until their use. Autofluorescence quenching was performed by treating the eyes with 50 mM NH4Cl in 0.1 M TBS for 30 min. Samples were blocked for an hour using 5% FBS and 0.3% triton X-100 diluted in 0.1 M TBS. When performing mouse on mouse staining, 1:25 mouse on mouse blocking reagent (Vector Laboratories, Burlingame, CA, USA) was added to the blocking. Primary antibodies diluted in the blocking solution were added after the blocking step and were incubated overnight at 4 °C (Table 2). Secondary antibodies were added after, diluted in blocking solution for four hours at room temperature (Table 2). A nuclear counterstain was performed using 5 µg/mL DAPI diluted in 0.1 M TBS for 10 min. Corneas were mounted using Vectashield Vibrance Mounting Medium (Vector Laboratories, Burlingame, CA, USA). All flat mount samples were imaged using Zeiss Axio Imager Z2 with an AxioCam MRc color CCD camera (Carl Zeiss, Oberkochen, Germany). To reduce non-specific autofluorescence, an unstained channel was imaged and used to subtract the fluorescence in the stained channels using Fiji software. Image processing and quantifications were performed using Fiji.

#### Cell cultures

HCECs were grown overnight on coverslips in 12-well cell culture plates to 80% confluence in KeratinoMAX medium (Wisent, St-Bruno, QC, Canada. For pre-treatments, cells were pre-exposed to GF19 (free peptide or encapsulated in SiNP) for 1 h after which cells in PBS and fixed with 4% paraformaldehyde for immunofluorescence after 48 h of culture. Antibody details are given in Table 2. Imaging was performed on Zeiss Confocal LS M880 upright multiphoton system. Image Analysis was performed using Fiji and Adobe Photoshop.

### Synthesis of silica nanoparticles and encapsulation of GF19

Chemicals such as ammonium hydroxide, cyclohexane, and tetraethyl orthosilicate (TEOS), were purchased from VWR International (Mississauga, Ontario, Canada). Triton X-100 detergent was acquired from Sigma-Aldrich. Ultra-pure water (Millipore Elix Essential Water Purification System) was used for all experiments.

GF19 was encapsulated within silica nanoparticles (SiNPs) as we described for its parent peptide, LL37^21^. In a round-bottomed flask, immersed in an oil bath at a temperature of 50 °C, a solution was prepared by dissolving 4 mL of Triton X-100 in 12 mL of cyclohexane. Next, a solution containing 4.8 mg of GF19 was dissolved in 2 mL of distilled water and subsequently combined with a cyclohexane-Triton X-100 solution in order to produce a reverse water-in-oil (w/o) microemulsion. Subsequently, a gradual addition of 1.5 mL of TEOS was performed, followed by the addition of 0.2 mL of ammonium hydroxide solution. The reactants underwent agitation for a duration of 48 h at a temperature of 50 ℃. SiNPs were initially treated with ethanol three times, followed by rinsing with distilled water and then lyophilization for the purpose of preservation.

#### Characterization of nanoparticles

Fourier-transform Infrared spectroscopy (FTIR-ATR) analysis using a Thermo Fisher Scientific Nicolet 6700 / Smart iTR device was performed to characterize the SiNPs. A TA Instruments DSC 25 was used to perform Differential Scanning Calorimetry (DSC) on the samples that were positioned within hermetically sealed pans made of aluminum-lead alloy. A scanning rate of 10 °C per minute was used. Zeta potential and particle size of the samples were measured on a ZetaView NTA device.

#### Encapsulation efficiency (EE) of SiNP-GF19

In order to determine the EE of the generated SiNP-GF19, the GF19 molecules were labeled with fluorescein isothiocyanate (FITC) to facilitate monitoring using a UV–Visible spectrophotometer. The subsequent equation was employed for the assessment of EE (%):

$$EE\left(\text{\%}\right)=\frac{Weight \;of \;GF19\in NPs}{Initial \;weight \;of \;GF19} \times 100$$

To achieve this objective, certain amount of each freeze-dried sample of NPs was dissolved in 1 mL of phosphate-buffered saline (PBS). After a period of 24 h, GF19 content was assessed using a UV–visible spectrophotometer (TECAN NanoQuant Plate™) at a wavelength of 454 nm^59,60^.

### GF19 interaction with HCECs

HCECs were grown overnight on 12-well chambered slides (Ibidi GmbH, Gräfelfing, Germany) to 80% confluence. The cells were infected with an MOI of 0.5 for one hour, after which the virus-containing media was removed. Fresh media was added, and cells were fixed in 4% paraformaldehyde for immunocytochemistry (n = 6 samples). Samples were blocked in 5% fetal bovine serum with 0.1% Triton X-100 diluted in 0.01 M PBS. FITC-tagged GF19 was used to visualise peptide-cell-virus interactions. Primary antibody (Table 2) was added in blocking solution and incubated with the samples overnight at 4 degrees. Secondary antibody was  applied for 1 h at room temperature after rinsing with PBS. Nuclei was counterstained with 5 µg/mL DAPI diluted in 0.01 M PBS for 10 min. Imaging was performed on Zeiss Confocal LS M880 upright multiphoton microscope system. Image analysis was performed using Fiji and Adobe Photoshop.

### Statistical analyses

GraphPad Prism 9.3.0 (GraphPad Software LLC., San Diego, CA, USA) was used for the statistical analyses. Before starting the statistical analyses, Grubbs test with a confidence interval of 95% and as critical value of Z of 1.715036468 was performed for all experiments. Two outliers were found and reported in Table S2. Statistical analysis for BMDC was done using a one-way ANOVA followed by a post-hoc Dunnett’s multiple comparisons with a confidence interval of 95%. Statistical analyses for biocompatibility and antiviral efficacy assays were done by two-way ANOVA, followed by post-hoc Tukey's multiple comparisons test with a confidence interval of 95% for each marker (GraphPad Prism 9.3.0, GraphPad Software LLC., San Diego, CA, USA).

### Supplementary Information

Supplementary Information.

## Data Availability

The raw data supporting the conclusions of this article will be made available by the corresponding author May Griffith (May.Griffith@umontreal.ca) upon reasonable request.
