# Supplementary material for: Modified host defence peptide GF19 slows TNT-mediated spread of corneal herpes simplex virus serotype I infection
Source: Sci Rep. 2024 Feb 19;14:4096. doi: 10.1038/s41598-024-53662-4 (PMC10876564; doi:10.1038/s41598-024-53662-4)
Supplement: Supplementary file 1 — Supplementary Information. [file 41598_2024_53662_MOESM1_ESM.pdf]

Supplementary Information

Modified Host Defence Peptide GF19 slows TNT-mediated spread of Corneal Herpes Simplex Virus Serotype I Infection

Neethi C Thathapudi[1-3, †], Natalia Callai Da Silva[1-3,†], Kamal Malhotra[1,2,§], Sankar Basu[4], Mozhgan Aghajanzadeh-Kiyaseh[1-3], Mostafa Zamani-Roudbaraki[1-3], Marc Groleau[1,2,5], Félix Lombard-Vadnais[1], Sylvie Lesage[1,5], May Griffith[1-3,*]

[1]Maisonneuve-Rosemont Hospital Research Centre, Montreal, Quebec H1T 2M4, Canada
[2]Department of Ophthalmology, Université de Montréal, Montreal, Quebec H3C 3J7, Canada
[3]Institute of Biomedical Engineering, Université de Montréal, Montreal, Quebec H3T 1J4, Canada
[4]Department of Microbiology, Asutosh College, (Affiliated with University of Calcutta), Kolkata 700026, India
[5]Département de Microbiologie, Infectiologie et Immunologie, Université de Montréal, Montreal, Quebec H3T 1J4, Canada

* Correspondence: may.griffith@umontreal.ca

† These authors contributed equally to this work

§ Present address: University of Ottawa Heart Institute, Ottawa, Ontario K1Y 4W7, Canada

Supplementary Tables

Table S1. Sequences of peptides used in the design and study.

Peptide	Sequence
LL37	LLGDFFRKSKEKIGKEFKRIVQRIKDFLRNLVPRTES
KR12	- -KRIVQRIKDFLR - - - - -
FK13	-FKRIVQRIKDFLR - - - - -
GF17	GFKRIVQRIKDFLRNLV - -
GF19	GFKRIVQRIKDFLRNLVKL
GI 20	GIKEFKRIVQRIKDFLRNLV- -
Scrambled GF19	GRKVVKRIQFRLDLLNFIK

Table S2. Grubb's test to determine outliers in biocompatibility tests.

Grubbs' test	sGF19 24h, 50 µM	GF17 48h, 10 µM
	Mean: 0.856250425	Mean: 0.973736262
	SD: 0.465583611	SD: 0.52522729
	# of values: 6	# of values: 6
	Outlier detected? Yes	Outlier detected? Yes
	Significance level: 0.05 (two-sided)	Significance level: 0.05 (two-sided)
	Critical value of Z: 1.715036468	Critical value of Z: 1.715036468
	detected Z: 1.785340052	detected Z: 1.769753686

Table S3. Post-hoc Tukey test after 2-way ANOVA to examine differences in biocompatibility among GF19, scrambled GF19 (sGF19), GF17 and LL37 at 24 hours post-treatment, as graphically represented in Figure 3A.

Tukey's multiple comparisons test	Mean Diff.	95.00% CI of diff.	Significant?	Adjusted P Value
GF19				
0 µM vs. 5 µM	-0.1707	-0.3709 to 0.02945	No	0.1343
0 µM vs. 10 µM	-0.08258	-0.2828 to 0.1176	No	0.7874
0 µM vs. 25 µM	0.08149	-0.1187 to 0.2817	No	0.7955
0 µM vs. 50 µM	0.3713	0.1711 to 0.5715	Yes	<0.0001
sGF19				
0 µM vs. 5 µM	0.09045	-0.1097 to 0.2906	No	0.7256
0 µM vs. 10 µM	0.08439	-0.1158 to 0.2846	No	0.7737
0 µM vs. 25 µM	0.11	-0.09017 to 0.3102	No	0.5553
0 µM vs. 50 µM	0.1692	-0.04073 to 0.3792	No	0.177
GF17				
0 µM vs. 5 µM	-0.03159	-0.2318 to 0.1686	No	0.9925
0 µM vs. 10 µM	0.02995	-0.1702 to 0.2301	No	0.9939
0 µM vs. 25 µM	0.07635	-0.1238 to 0.2765	No	0.8316
0 µM vs. 50 µM	0.3182	0.1180 to 0.5184	Yes	0.0002
LL37				
0 µM vs. 5 µM	-0.137	-0.3372 to 0.06319	No	0.3293
0 µM vs. 10 µM	-0.1369	-0.3371 to 0.06328	No	0.33
0 µM vs. 25 µM	0.3548	0.1546 to 0.5550	Yes	<0.0001
0 µM vs. 50 µM	0.642	0.4418 to 0.8422	Yes	<0.0001

Table S4. Post-hoc Tukey test after 2-way ANOVA to examine differences in biocompatibility among GF19, scrambled GF19 (sGF19), GF17 and LL37 at 48 hours post-treatment as graphically represented in Figure 3A.

Tukey's multiple comparisons test	Mean Diff.	95.00% CI of diff.	Significant?	Adjusted P Value
GF19				
0 µM vs. 5 µM	-0.1751	-0.3753 to 0.02512	No	0.1175
0 µM vs. 10 µM	-0.1481	-0.3483 to 0.05211	No	0.2526
0 µM vs. 25 µM	-0.03845	-0.2386 to 0.1617	No	0.9843
0 µM vs. 50 µM	0.2969	0.09667 to 0.4971	Yes	0.0006
sGF19				
0 µM vs. 5 µM	-0.08223	-0.2824 to 0.1180	No	0.7901
0 µM vs. 10 µM	-0.1121	-0.3123 to 0.08809	No	0.5368
0 µM vs. 25 µM	-0.01867	-0.2189 to 0.1815	No	0.999
0 µM vs. 50 µM	0.01916	-0.1810 to 0.2194	No	0.9989
GF17				
0 µM vs. 5 µM	-0.1145	-0.3147 to 0.08565	No	0.5151
0 µM vs. 10 µM	-0.05392	-0.2639 to 0.1560	No	0.9547
0 µM vs. 25 µM	-0.1301	-0.3303 to 0.07013	No	0.3831
0 µM vs. 50 µM	0.06065	-0.1395 to 0.2608	No	0.9197
LL37				
0 µM vs. 5 µM	-0.09961	-0.2998 to 0.1006	No	0.6477
0 µM vs. 10 µM	-0.09818	-0.2984 to 0.1020	No	0.6602
0 µM vs. 25 µM	0.4833	0.2831 to 0.6835	Yes	<0.0001
0 µM vs. 50 µM	0.7554	0.5553 to 0.9556	Yes	<0.0001

Table S5. Post-hoc Tukey test after 2-way ANOVA to examine differences in antiviral activity among GF19, scrambled GF19 (sGF19), GF17 and LL37 at 24 hours post-treatment as graphically represented in Figure 4A.

Tukey's multiple comparisons test	Mean Diff.	95.00% CI of diff.	Significant?	Adjusted P Value
GF19				
0 vs. 5	0.7341	-0.4664 to 1.935	No	0.5075
0 vs. 10	1.551	0.3503 to 2.751	Yes	0.004
0 vs. 15	1.912	0.7111 to 3.112	Yes	0.0002
0 vs. 25	2.727	1.526 to 3.927	Yes	<0.0001
0 vs. 35	3.574	2.374 to 4.775	Yes	<0.0001
0 vs. 45	4.917	3.716 to 6.117	Yes	<0.0001
sGF19				

0 vs. 5	0.4079	-0.7926 to 1.608	No	0.9423
0 vs. 10	1.136	-0.06409 to 2.337	No	0.0748
0 vs. 15	1.248	0.04729 to 2.448	Yes	0.0366
0 vs. 25	1.854	0.6535 to 3.055	Yes	0.0003
0 vs. 35	1.386	0.1852 to 2.586	Yes	0.0139
0 vs. 45	1.939	0.7386 to 3.140	Yes	0.0001
GF17				
0 vs. 5	0.3019	-0.8986 to 1.502	No	0.9869
0 vs. 10	1.063	-0.1377 to 2.263	No	0.1153
0 vs. 15	1.776	0.5758 to 2.977	Yes	0.0006
0 vs. 25	2.713	1.512 to 3.913	Yes	<0.0001
0 vs. 35	3.307	2.107 to 4.508	Yes	<0.0001
0 vs. 45	4.402	3.201 to 5.602	Yes	<0.0001
LL37				
0 vs. 5	1.713	0.5124 to 2.913	Yes	0.0011
0 vs. 10	3.313	2.112 to 4.513	Yes	<0.0001
0 vs. 15	3.12	1.920 to 4.321	Yes	<0.0001
0 vs. 25	4.917	3.716 to 6.117	Yes	<0.0001
0 vs. 35	3.645	2.302 to 4.987	Yes	<0.0001
0 vs. 45	4.525	3.324 to 5.725	Yes	<0.0001

Table S6. Post-hoc Tukey test after 2-way ANOVA to examine differences in antiviral activity among GF19, scrambled GF19 (sGF19), GF17 and LL37 at 48 hours post-treatment as graphically represented in Figure 4A.

Tukey's multiple comparisons test	Mean Diff.	95.00% CI of diff.	Significant?	Adjusted P Value
GF19				
0 vs. 5	0.5423	-1.241 to 2.326	No	0.9661
0 vs. 10	0.7488	-1.035 to 2.532	No	0.8564
0 vs. 15	0.9173	-0.8661 to 2.701	No	0.6997
0 vs. 25	1.621	-0.1626 to 3.404	No	0.0981
0 vs. 35	2.476	0.6925 to 4.259	Yes	0.0015
0 vs. 45	5.965	4.182 to 7.749	Yes	<0.0001
sGF19				
0 vs. 5	0.7252	-1.058 to 2.509	No	0.8739
0 vs. 10	0.8382	-0.9452 to 2.622	No	0.7794
0 vs. 15	0.8691	-0.9143 to 2.653	No	0.7493
0 vs. 25	0.9549	-0.8285 to 2.738	No	0.6592
0 vs. 35	1.677	-0.1068 to 3.460	No	0.0784

0 vs. 45	1.554	-0.2290 to 3.338	No	0.1266
GF17				
0 vs. 5	0.3738	-1.410 to 2.157	No	0.9951
0 vs. 10	0.4958	-1.288 to 2.279	No	0.9782
0 vs. 15	0.7528	-1.031 to 2.536	No	0.8533
0 vs. 25	1.098	-0.6856 to 2.881	No	0.5002
0 vs. 35	1.945	0.1620 to 3.729	Yes	0.024
0 vs. 45	4.091	2.307 to 5.874	Yes	<0.0001
LL37				
0 vs. 5	0.9727	-0.8107 to 2.756	No	0.6396
0 vs. 10	2.184	0.4004 to 3.967	Yes	0.0074
0 vs. 15	3.295	1.512 to 5.079	Yes	<0.0001
0 vs. 25	5.872	4.089 to 7.656	Yes	<0.0001
0 vs. 35	5.3	3.517 to 7.084	Yes	<0.0001
0 vs. 45	6.162	4.379 to 7.946	Yes	<0.0001

Supplementary Figure

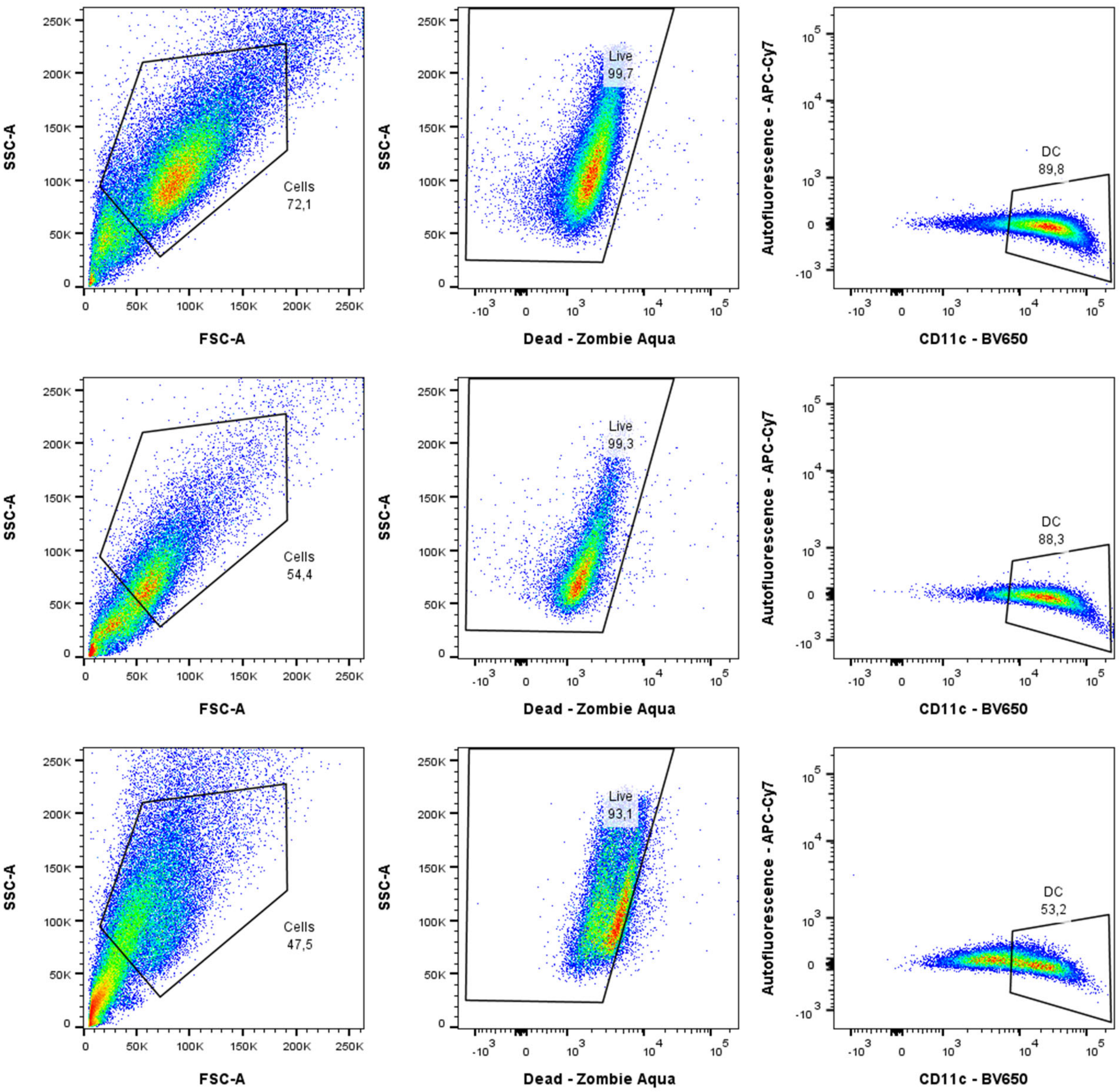

Figure S1. Gating strategy for BMDC flow cytometry study of the effect of GF19 treatment on BMDC activation. The effects of GF19 were compared to LPS that served as a positive control for activation of BMDCs, as shown in Figure 3B. The BMDCs were gated as CD11c^{hi} viable single cells.
